# A randomized controlled trial comparing trauma-focused treatment with and without concurrent personality disorder treatment in patients with posttraumatic stress disorder and comorbid borderline personality disorder

**DOI:** 10.1192/j.eurpsy.2024.1385

**Published:** 2024-08-27

**Authors:** A. Snoek, J. Dekker, A. Beekman, A. Beekman, I. Aarts, I. Aarts, A. van den End, M. Blankers, C. Vriend, O. van den Heuvel, K. Thomaes

**Affiliations:** ^1^Sinai Centrum, Arkin, Amstelveen; ^2^Arkin; ^3^Psychiatry, Amsterdam UMC; ^4^Sinai Centrum, Arkin, Amsterdam, Netherlands

## Abstract

**Introduction:**

Posttraumatic stress disorder (PTSD) and borderline personality disorder (BPD) often co-occur. There is growing motivation among clinicians to offer trauma-focused treatments, such as Eye Movement Desensitization and Reprocessing (EMDR), to patients with PTSD and comorbid BPD. However, a large subgroup of these patients does not sufficiently respond to trauma-focused treatment and is more likely to be excluded or dropout from treatment. Dialectical Behaviour Therapy (DBT) for BPD is well established and although there is some evidence that DBT combined with prolonged exposure is twice as effective in reducing PTSD symptoms than DBT alone, the comparative efficacy of trauma-focused treatment with and without concurrent PD treatment has not been investigated yet.

**Objectives:**

The current study will therefore evaluate the comparative clinical efficacy of EMDR with and without concurrent DBT in patients with PTSD and comorbid BPD.

**Methods:**

Adult patients were randomly assigned to EMDR with (*n* = 63) or without concurrent DBT (n = 63). A wide range of clinician-administered and self-report assessments were conducted before, during and up to six months after treatment. The longitudinal change in PTSD severity as the primary outcome was measured using multilevel mixed regression in SPSS. The present study is part of the overarching Prediction and Outcome Study in comorbid PTSD and Personality Disorders (PROSPER), which consists of a second RCT comparing trauma-focused treatment with and without concurrent PD treatment in patients with PTSD and cluster C PD.

**Results:**

Results, available in January 2024, will reveal which treatment works best for this difficult-to-treat group of patients.

**Conclusions:**

This is the first study to compare the clinical efficacy of EMDR with and without concurrent DBT in patients with PTSD and comorbid BPD. Results will reveal which treatment works best for this difficult-to-treat group of patients.

**Disclosure of Interest:**

None Declared

